# Intraoperative positioning system-guided antegrade in situ laser fenestration in an aortic model

**DOI:** 10.1016/j.jvscit.2026.102124

**Published:** 2026-01-06

**Authors:** Emily Burnett, Emidio Germano, Allie Olmstead, Benjamin Samberg, Robert Meisner, Hannah Hofacker, Michael Bronez, Vikash Goel, Animesh Rathore

**Affiliations:** a Macon and Joan Brock Virginia Health Sciences Eastern Virginia Medical School at Old Dominion University, Norfolk, VA; b Division of Vascular Surgery, Department of Surgery, Lankenau Heart Institute, Main Line Health, Wynnewood, PA; c Department of Innovation and Research, Centerline Biomedical, Cleveland, OH

**Keywords:** Aortic aneurysm, Thoracoabdominal aneurysm, Complex aortic aneurysm repair, In situ laser fenestration, IOPS-guided fenestration

## Abstract

**Objective:**

Fenestrated endovascular aneurysm repair (fEVAR) is routinely being used for visceral vessel incorporation in complex aneurysmal pathology; however, it is associated with prolonged procedure times, high radiation exposure, high contrast use, and multiple staged procedures. To combat these limitations, Centerline Biomedical developed the three-dimensional surgical navigation and visualization technology: intraoperative positioning system (IOPS). We previously presented our IOPS-guided antegrade in situ laser fenestration in a standard aortic model. This case report details our experience using IOPS guidance during a fEVAR procedure in a representative thoracoabdominal aortic aneurysm phantom model.

**Methods:**

A silicone thoracoabdominal aortic segment was three-dimensional printed based on high-resolution computed tomography angiogram imaging of a patient with thoracoabdominal aortic aneurysm. An experimental fEVAR procedure was performed on the phantom through simulated vascular entry points in a heated water bath to simulate ambient body temperature. A thoracic endograft was deployed within the phantom, followed by in situ laser fenestrations using intraoperative positioning system guidance for each visceral vessel. Bridging stents were placed through the created fenestrations into the respective visceral vessels. Except for cannulation confirmation and completion images, there was minimal to no radiation used.

**Results:**

Stent deployment was confirmed via a single shot radiograph. Cannulation times were 45 seconds for right renal artery, 96 seconds for the celiac artery, 51 seconds for the superior mesenteric artery, and 687 seconds for the left renal artery. Completion angiogram demonstrated widely patent renal arteries, celiac artery, superior mesenteric artery, and inferior mesenteric artery with accurately aligned stent grafts.

**Conclusions:**

This benchtop phantom model demonstrates early feasibility of IOPS-guided in situ laser fenestration as a promising radiation-sparing technique with the added benefit of obviating a prestenting procedure and expediting fEVAR procedure times. However, research should be conducted to further evaluate the safety and efficacy of IOPS application in fEVAR in humans.

**Article Highlights**

• **Type of Research:** Benchtop phantom model
• **Key Findings:** An experimental fenestrated endovascular aneurysm repair procedure was performed on a three-dimensional representative aortic segment, using intraoperative positioning system guidance for in situ four-vessel laser fenestration and cannulation. Cannulation times were 45 seconds for the right renal artery, 96 seconds for celiac artery, 51 seconds for the superior mesenteric artery, and 687 seconds for the left renal artery. Completion angiogram demonstrated widely patent renal arteries, celiac artery, superior mesenteric artery, and inferior mesenteric artery with accurately aligned stent grafts.
• **Take Home Message:** This benchtop phantom model demonstrates early feasibility of intraoperative positioning system-guided in situ laser fenestration as a promising radiation-sparing technique with the added benefit of obviating a prestenting procedure and expediting fenestrated endovascular aneurysm repair procedure times.

Fenestrated endovascular aneurysm repair (fEVAR) is a technique used to treat thoracoabdominal aortic aneurysm (TAAA) or abdominal aortic aneurysms (AAAs) without an infrarenal aortic neck suitable for a classic EVAR procedure.^1^ By creating fenestrations within the main endograft, this technique allows for a less invasive approach to complex aortic disease, notably in patients deemed too high risk for open AAA repair.^2^^,^^3^

In response to the growing demand for fenestrated aortic repair, devices such as the Cook Zenith Fenestrated endograft have been developed.^4^^,^^5^ Additionally, physician-modified endografts offer an off-label approach, allowing surgeons to customize off-the-shelf endografts with hand-made fenestrations tailored to a patient's anatomy.^6^^,^^7^ Another innovative off-label method, in situ laser fenestration (ISLF), uses a laser catheter to create fenestrations intraoperatively to accommodate visceral vessels.^8^ This technique typically involves a separate prestenting intervention of all target vessels before the deployment of the stent graft in the aorta to serve as targets for ISLF creation. During fEVAR with ISLF, an endograft is placed initially covering the ostia of target vessels, followed by the creation of antegrade ISLF with the aid of multiple orthogonal views, and cannulation and placement of a bridging stent through the fenestration into the target vessels.

These advancements have significantly broadened the applicability of fEVAR; however, they require significant operator expertise and precision, which restricts their wider adoption. Key challenges include the risk of location mismatch between fenestrations and the ostia of the target visceral branches leading to visceral ischemia, as well as potential complications such as retroperitoneal perforation and bleeding.^9^^,^^10^ Additionally, achieving accurate alignment of fenestrations with critical abdominal branch vessels often requires extended fluoroscopy, resulting in increased radiation exposure for both operators and patients, along with greater use of iodinated contrast agents.^11^^,^^12^

Centerline Biomedical developed an intraoperative positioning system (IOPS) as an adjunct to fluoroscopy in order to address limitations with respect to visualization, navigation, radiation safety, and dependence on contrast media. This system provides real-time, three-dimensional (3D) image guidance based on preacquired high-resolution computed tomography angiograms (CTAs), allowing IOPS catheters and guidewires to be navigated precisely with 3D color visualization without angiography or fluoroscopy. This report presents a proof-of-concept study using IOPS guidance during a four-vessel laser fenestration EVAR procedure in a representative AAA phantom model.

## Methods

**Fig 1:**
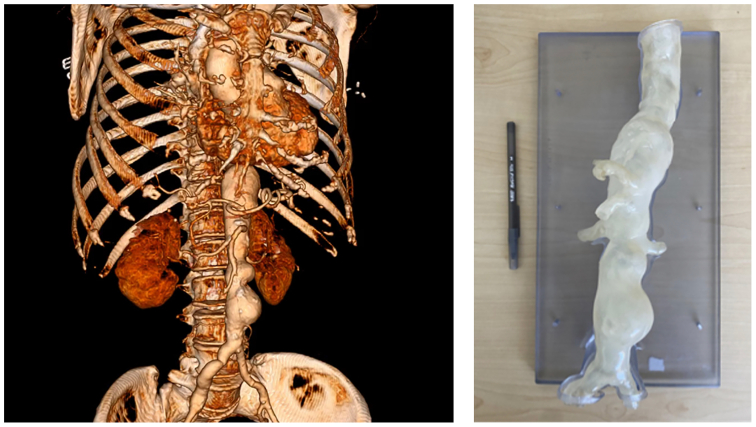
Patient preoperative computed tomography angiogram (CTA) (*left*) used to 3D print the silicone phantom of the aortic segment (*right*).

Our team used the IOPS in a representative phantom model of a TAAA to carry out a fEVAR using in situ four-vessel laser fenestration on a Cook Zenith Alpha endograft. This study was experimental, with a silicone abdominal aortic model that was 3D printed based on a preoperative high-resolution CTA of a patient with a TAAA (Fig 1). This study did not require institutional review board approval because it did not involve direct patient interaction and intervention. The operator was an attending surgeon at our institution whose experience with ISLF is in urgent and emergent indications with reference to McKinley et al for technique.^8^ We do not have IOPS for clinical use at our institution, so our operator had no prior experience using IOPS for either ISLF or conventional fenestration.

The experiment was performed in an endovascular intervention suite equipped with a ceiling-mounted Philips Azurion 7 M20 fluoroscopic device. The aortic phantom was placed inside of a circulating water bath tank heated to 98°F and covered with an opaque black tarp to protect the operators against potential injuries from the laser catheter during fenestration.

The IOPS setup included an electromagnetic field generator directly under the operating table, as well as a sensor interface unit underneath the foot of the table. In clinical use, an IOPS fiducial patch is typically placed on the patient's back. In this simulation, we placed the patch under the water bath directly below the phantom. A cone-beam CT was performed. This image was then fused with the patient's preoperative CTA in the IOPS system for image coregistration with the 3D aortic map generated from the CTA.

**Fig 2:**
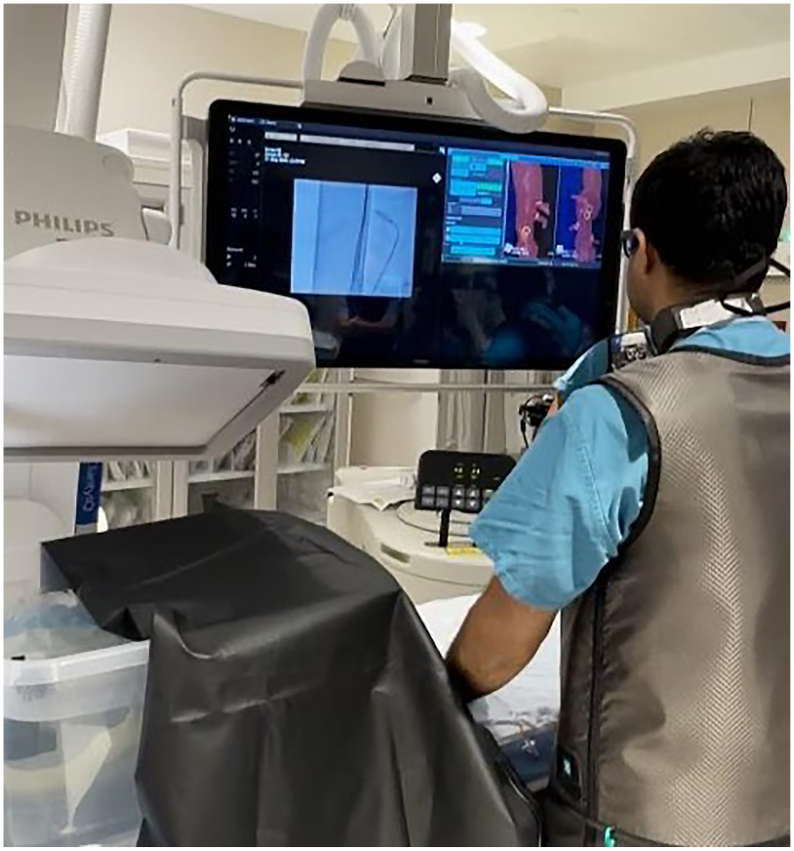
Intraoperative positioning system (IOPS) and fluoroscopy display during the experiment.

**Fig 3:**
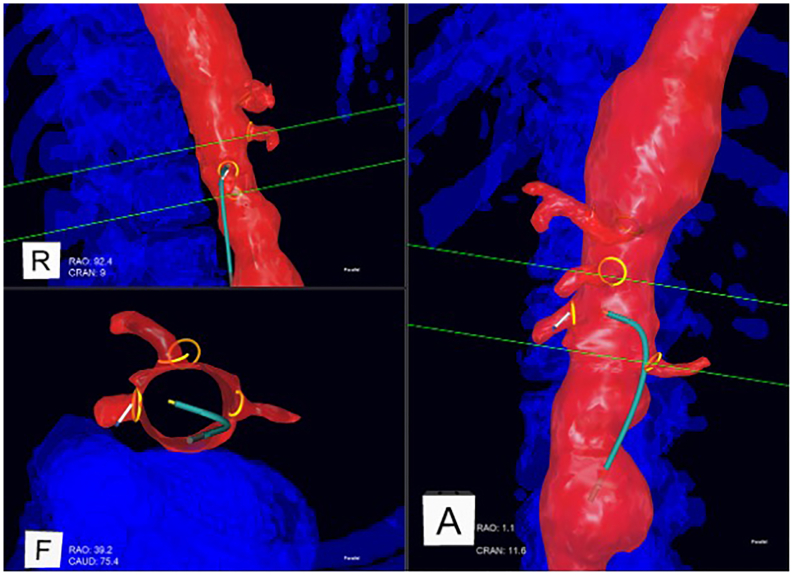
Intraoperative positioning system (IOPS) navigation during selection of the right renal artery (RRA). The catheter is shown in *cyan* and the guidewire tip in *white*. Up to four simultaneous projections are available (three shown). The *lower left* demonstrates an endoluminal view.

The IOPS display was placed on the large procedure room display adjacent to the fluoroscopy image (Fig 2). Up to four simultaneous projections were available, including an endoluminal view of the interior of the vessel lumen (Fig 3).

An experimental ISFL procedure was performed targeting the celiac artery (CA), superior mesenteric artery (SMA), right renal artery (RRA), and left renal artery (LRA). The primary end points were technical success and cannulation time. Technical success was defined as correctly aligned and patent stent grafts post procedure. The cannulation time for each visceral was defined as the time after the IOPS imaging was lined up with the target vessel from the initial wire/catheter movement to when the wire/catheter entered the target vessel after the fenestration was created.

**Fig 4:**
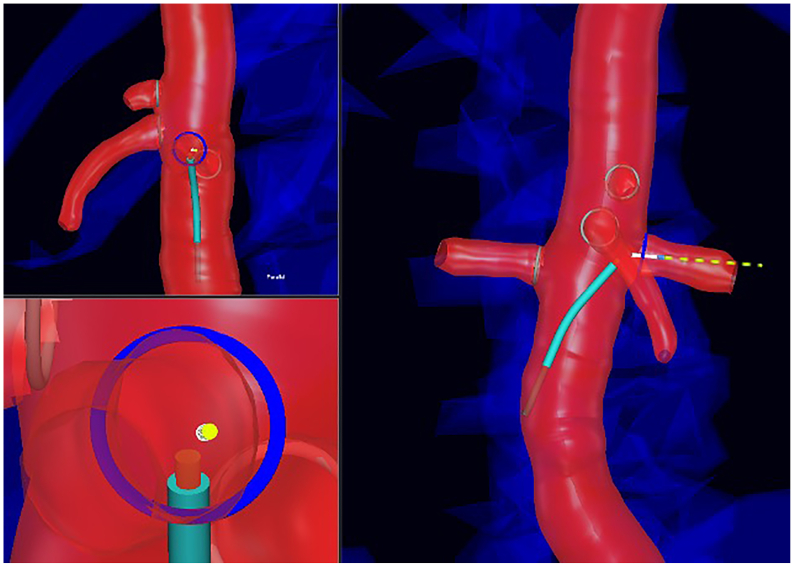
Example of center ostium and directional indicator functions being used in cannulation of a left renal artery (LRA). Target ostium in *blue*, guidewire tip in *white*, directional indicator in *yellow-green*. (*Top left*) En face view of target ostium, with directional indicator foreshortened to almost a single dot. (*Right*) En profil view of target ostium, with directional indicator at maximum length. (*Bottom left*) Higher magnification version of the top left projection.

Two navigational functions of the IOPS software were leveraged to assist with each laser fenestration task. First, the center ostium feature was used to automatically align each visualization projection to be orthogonal or parallel to the ostium of the target visceral vessel, providing optimized viewing angles for the cannulation. Second, the directional indicator feature was used to provide an animated series of dots emanating from the virtual representation of the guidewire. When the guidewire tip is correctly oriented perpendicular to the target vessel ostium, the en profil projection would show the directional indicator at its greatest length, whereas the en face projection would show it foreshortened almost to a single dot (Fig 4).

## Results

**Fig 5:**
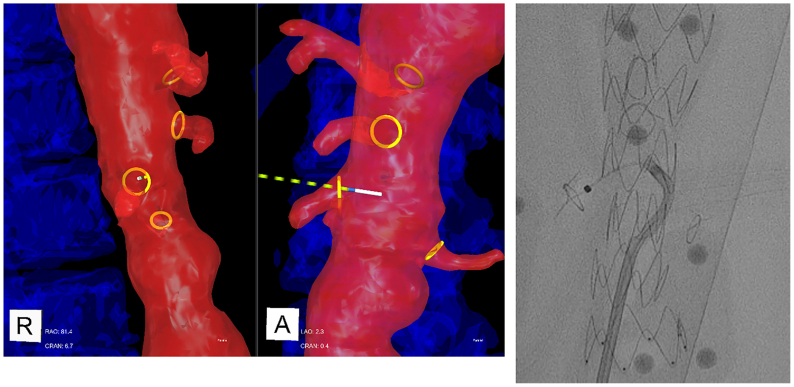
Intraoperative positioning system (IOPS) navigation (*left*) and confirmation angiogram (right) demonstrating position of IOPS guidewire inside right renal artery (RRA) confirming fenestration position. IOPS fiducial markers are visible in the radiograph.

We conducted an initial check using a Medtronic TourGuide 7F 65-cm steerable sheath and IOPS 0.035″ guidewire to access all four visceral branches: the RRA, LRA, SMA, and CA. The Cook Zenith Alpha endograft was then deployed in the phantom under fluoroscopy. We used a Medtronic TourGuide 7F 65-cm steerable sheath, a Philips Turbo-Elite 2.3-mm OTW 0.035″ atherectomy laser, and an IOPS 0.035″ guidewire to create the fenestrations. It is our institutional practice to use a Philips Turbo-Elite 2.3 mm OTW 0.035″ for in situ fenestration. Starting with the RRA, and using the IOPS biplane projections, we targeted the IOPS ostium marker with maximal foreshortening of the directional indicator from the en face view. When the IOPS showed the correct position at the RRA, the laser was energized to create a fenestration. The IOPS guidewire was advanced into the branch vessel, confirming correct fenestration positioning. The laser catheter was then advanced into the branch, the IOPS guidewire was removed, and angiography was performed to verify fenestration (Fig 5).

**Fig 6:**
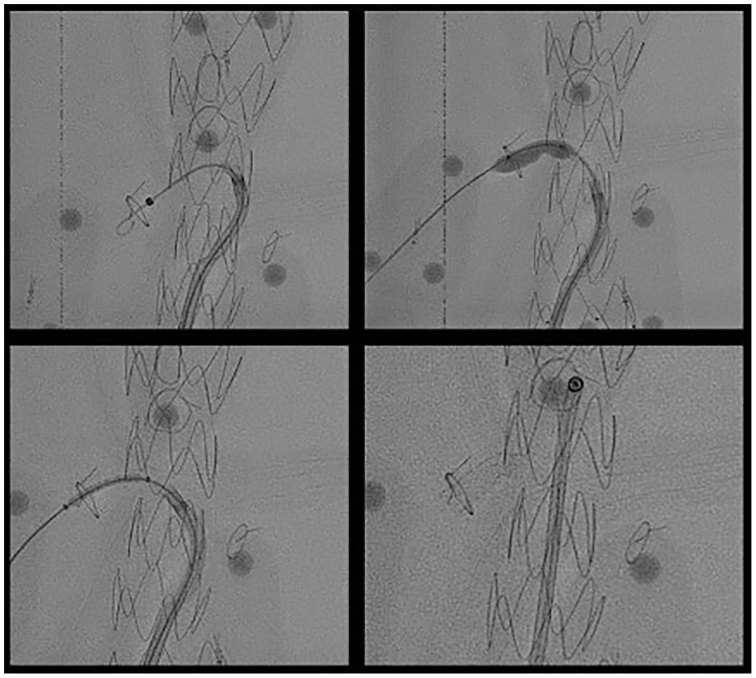
(*Top left*) Rosen guidewire advanced through fenestration. (*Top right*) Fenestration expanded via balloon angioplasty. (*Bottom left*) iCast bridging stent graft advanced through fenestration. (*Bottom right*) Bridging stent graft implanted.

**Fig 7:**
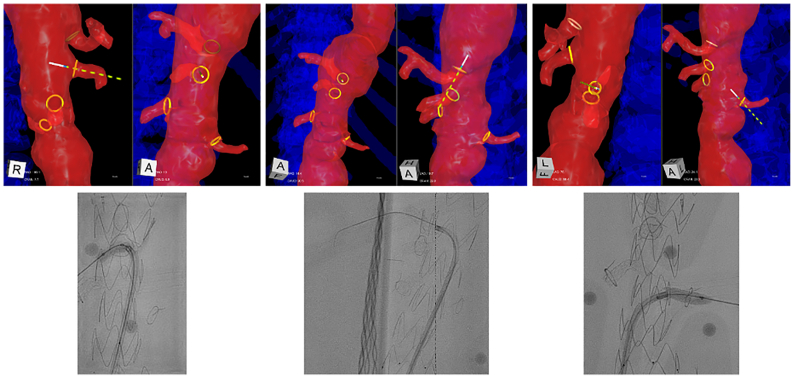
Intraoperative positioning system (IOPS) guidance was used to position the laser filament for fenestrations of superior mesenteric artery (SMA), celiac trunk, and left renal artery (LRA), and the fenestrations were ballooned and bridging stents placed under fluoroscopy.

A stiff Rosen guidewire was advanced into the RRA to support a balloon and stent, and we performed an additional three to four passes with the laser to ensure adequate fenestration. We then performed percutaneous balloon angioplasty to expand the fenestration and a sheath was advanced. Finally, we deployed a bridging balloon-expandable Atrium iCast stent graft in the RRA (Fig 6). This process was repeated for the SMA, CA, and LRA in that order (Fig 7). Cannulation times are shown in the Table.

**Table:** Laser fenestration cannulation times for each visceral vessel

Visceral vessel	Cannulation time
RRA	45 seconds
CA	1 minutes 36 seconds
SMA	51 seconds
LRA	11 minutes 27 seconds

*CA,* celiac artery; *LRA,* left renal artery; *RRA,* right renal artery; *SMA,* superior mesenteric artery.

**Fig 8:**
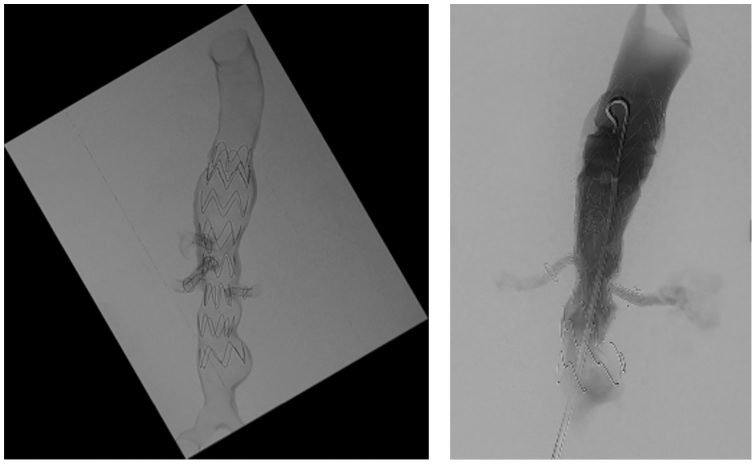
Completion single shot radiograph confirming stent deployment in all four visceral vessels (*left*) and completion angiogram demonstrating vessel patency (*right*).

Stent deployment was confirmed via single shot radiography (Fig 8). Completion angiogram demonstrated widely patent renal arteries, CA, and SMA with accurately aligned stent grafts. Completion cone-beam CT further confirmed accurately aligned-aligned stent grafts.

## Discussion

In high-risk patients who lack a suitable aortic neck for a landing zone below the renal arteries, fEVAR is a viable and life-saving alternative to open repair for AAA. However, it is the vascular intervention associated with the highest radiation exposure.^11^ As a multistaged procedure, fEVAR involves significantly more steps than traditional EVAR, resulting in substantially longer procedural times and increased radiation exposure.^13^ A multicenter survey from the UK reported a median fluoroscopy time of 24.8 minutes for standard EVAR, compared with 61.7 minutes for fEVAR.^14^ Additionally, Kirkwood et al^11^ observed escalating patient radiation doses with procedural complexity: four-vessel fEVARs accounting for 3020 mGy, compared with 2670 mGy for three-vessel and 1600 mGy for two-vessel cases. Operator radiation exposure showed a similar trend, with four-vessel fEVARs associated with doses of 440 μSv, compared with 170 μSv for three-vessel procedures.^11^

Numerous institutional studies and specialty guidelines^15^ have highlighted the occupational health risks associated with ionizing radiation exposure, including an increased risk of cataract formation^16^ and solid organ cancers,^17^^,^^18^ Prolonged procedure times during fEVAR have also been associated with adverse events such as renal dysfunction, mesenteric ischemia, and mortality.^19^, ^20^, ^21^, ^22^ These findings reinforce the critical need to mitigate risk by optimizing standard endovascular techniques.

Designed to enhance accuracy and efficiency in endovascular interventions, Centerline Biomedical developed the IOPS, an advanced endovascular navigation platform. The integration of a 3D vessel map generated from preoperative CT imaging with real-time electromagnetic tracking enabled by proprietary wires and catheters offers a significant potential for simplifying navigation in complex endovascular repairs. We were able to successfully use this system in a proof-of-concept study to perform a fEVAR with ISLF. Although angiography was used to confirm branch vessel cannulation, radiation exposure was notably limited, with fluoroscopy time reduced to <40 minutes. To our best knowledge, our study is the first to demonstrate the feasibility of using the IOPS as primary guidance for ISFL in fEVAR.

Hoell et al^23^ have presented a study showing safety and feasibility of IOPS image guidance, but that investigation stopped short of quantifying potential benefits as compared with fluoroscopy alone. Muluk et al^24^ presented a case series in which three out of four patients with chronic mesenteric ischemia successfully underwent IOPS-guided CA and SMA interventions. The study highlighted, however, one case of mismatch between the displayed location in the aortic model with the actual position of the wire and catheter on angiogram, which was attributed to shifts in the trackpad's position during the procedure. Cannulation times in their series ranged from 45 to 90 seconds with IOPS guidance, markedly shorter than the 7 minutes required in traditional mesenteric intervention.^24^ Farivar^25^^,^^26^ reported a similar case experience with a 39-second IOPS-guided SMA cannulation as well of IOPS’ clinical utility in mapping structures such as true and false lumens.

In our phantom model, the cannulation times ranged from 45 seconds for the RRA and 11 minutes and 27 seconds for the LRA. The prolonged cannulation time for the LRA was mainly due to its steep angulation, which posed challenges in achieving an optimal angle for the puncture and catheter advancement into the vessel. Additionally, although the cannulation times were part of the end points studied, rather than focusing on expeditious visceral cannulation attempts, a significant amount of time was spent at each step on educating trainees and personnel involved in the procedure, which certainly impacted the overall cannulation times. The aim of this study was to serve as a proof of concept on a clinically accurate model. Future investigations are warranted to compare cannulation times with baseline and evaluate the learning curves of attendings, residents, and students when using IOPS.

Typically, when performing antegrade ISLF, it is necessary to prestent the target branch vessels. The stents provide the only means of inferring the vessels' positions and orientations because it is not possible to infuse them with contrast media before fenestrating the graft fabric. This prestenting procedure represents complexity, cost, and patient risk. In this experiment, because the IOPS vessel map allowed appreciation of the branch vessels' geometry in 3D, we were able to reach each target vessel without the need for prestenting.

Since this in vitro work, collaborators at other institutions have described some early clinical experience using IOPS image guidance in two ISLF procedures. In both cases, the target branch vessels were prestented to support fluoroscopic navigation and mirror clinical practice. In one case, a thoracic EVAR stent graft was deployed to seal off the aneurysm sac using a standard technique with the renal arteries covered as a part of the treatment plan. A full arterial map was visible using the IOPS. This map included notations outlining the renal arteries, supporting visualization of the vessels and their ostia despite being their covered by the device. A steerable sheath and an IOPS guidewire were used to navigate to the level of the renal artery. The sheath was positioned at the location of the desired fenestration and aligned with the target vessel by referencing the IOPS guidewire and vessel map. The IOPS guidewire was exchanged for a 1.7-mm Philips Turbo-Elite laser catheter and a 0.018″ guidewire that were used to create a fenestration using the ISLF technique. The laser catheter was removed, and fluoroscopic guidance was used to deliver a balloon-expandable covered stent and confirm aneurysm sealing and patency of the branch. The process was performed for both renal arteries. Fluoroscopy and IOPS times were 1 minute and 7 minutes, respectively, for the RRA and 4 minutes and 10 minutes for the LRA. Although this clinical workflow differs from the techniques used in this study, the experience suggests clinical feasibility and also suggests that minimization of radiation exposure time is possible.

Ultimately, IOPS offers the potential for financial advantages by eliminating the need for the separate prestenting procedure and reducing the procedure times required in fEVAR, thereby decreasing operating room costs.^27^ These savings could help to offset the expenses of integrating this technology into clinical workflows.^28^ The IOPS products—the sterile single-use devices, the capital equipment, and the software—are marketed by Centerline Biomedical. There is typically a fixed cost to procure the system and recurring costs dependent on use similar to other equipment such as intravascular ultrasound. The capital equipment is compatible with all major fixed angiography systems, and one system can be moved from room to room as needed. The company has a variety of procurement models to accommodate differing budget needs. At this stage in the company's product launch, pricing is negotiated on a case-by-case basis. Nonetheless, in vivo studies comparing IOPS-guided fEVAR with conventional techniques are essential to comprehensively evaluate its effects on patient outcomes and the safety of health care personnel. When paired with ongoing advancements in procedural protocols, IOPS holds the promise of establishing a new benchmark for safer and more efficient endovascular repair.

## Conclusions

This benchtop model underscores the potential of IOPS guidance in FEVAR, demonstrating its ability to decrease patient and health care personnel radiation exposure and shorten procedure times. Although these initial findings are promising, further studies involving human models are essential to confirm the clinical efficacy and safety of IOPS guidance in this application. A progressive study approach, first using conventional ISLF workflows and transitioning toward the elimination of prestenting, is called for. This study provides early evidence of feasibility, offering a foundation for transformative advancements in fEVAR and improving patient outcomes in complex endovascular interventions.

### Limitations

Our study has several limitations, the first one being the use of a phantom model to represent a human aorta. The more rigid nature of the phantom makes it less prone to conformational changes. Further studies need to delineate anatomical changes after stiff wires, catheter, and endograft insertion into the aorta, which may create inaccuracies between the position represented in the 3D virtual map and the actual aorta. This experiment only evaluated the feasibility of performing the laser fenestration portion of the intervention. It would be worthwhile to evaluate the impact IOPS may have on completing the repair, including the bifurcated abdominal graft. As with every new technology, a learning curve exists on its use and interpretation, which may affect procedural times and early outcomes.

## Author contributions

Conception and design: HH, MB, VG, AR

Analysis and interpretation: EG, AO, RM, VG, AR

Data collection: EB, AO, BS, MB, VG, AR

Writing the article: EB, EG, AO, BS, VG, AR

Critical revision of the article: EB, EG, AO, RM, HH, MB, VG, AR

Final approval of the article: EB, EG, AO, BS, RM, HH, MB, VG, AR

Statistical analysis: Not applicable

Obtained funding: Not applicable

Overall responsibility: AR

## Funding

This research received a grant from Centerline Biomedical to cover the Article Processing Charge for publication.

## Disclosures

V.G. is the CTO and founder of Centerline Biomedical. A.R. is a consultant for Medtronic.
